# Effect of glucocorticoids on expression of cutaneous antimicrobial peptides in northern leopard frogs (*Lithobates pipiens*)

**DOI:** 10.1186/s12917-015-0506-6

**Published:** 2015-08-08

**Authors:** Laetitia Tatiersky, Louise A. Rollins-Smith, Ray Lu, Claire Jardine, Ian K. Barker, Mary Ellen Clark, Jeff L. Caswell

**Affiliations:** Department of Pathobiology, University of Guelph, Guelph, ON N1G 2W1 Canada; Departments of Pathology, Microbiology and Immunology and of Pediatrics, Vanderbilt University School of Medicine, Nashville, TN 37232 USA; Department of Biological Sciences, Vanderbilt University, Nashville, TN 37232 USA; Department of Molecular and Cellular Biology, University of Guelph, Guelph, ON N1G 2W1 Canada

**Keywords:** Chytridiomycosis, Antimicrobial peptides, Skin, Frogs, Corticosteroid, Quantitative RT-PCR

## Abstract

**Background:**

Many species of frogs secrete cutaneous antimicrobial peptides that are capable of killing *Batrachochytrium dendrobatidis*. Some of these species are nonetheless susceptible to chytridiomycosis, suggesting that host factors causing dysregulation of this innate immune response may be important in pathogenesis. Since stresses, such as from environmental perturbations, are a potential cause of such dysregulation, this study investigated the effect of glucocorticoid on cutaneous gene expression of these antimicrobial peptides.

**Results:**

Northern leopard frogs (*Lithobates pipiens*) were injected with either the corticosteroid methylprednisolone or saline every 48 h. Norepinephrine-elicited cutaneous secretions were collected every 8 days for 40 days. Gene expression of antimicrobial peptides (brevinin-1P and ranatuerin-2P) in the cutaneous secretions was measured relative to the reference genes EF1-α and RPL8 using quantitative RT-PCR. Corticosteroid treatment was associated with a significant increase in brevinin-1P gene expression, which was most notable at 24–40 days of corticosteroid administration. Ranatuerin-2P expression followed a similar but non-significant trend.

**Conclusion:**

This treatment protocol, including corticosteroid-administration and frequent norepinephrine-induced secretion, increased AMP gene expression in the skin of *L. pipiens* under these experimental conditions. The findings do not support the hypothesis that environmental stress predisposes frogs to chytridiomycosis by causing glucocorticoid-induced suppression of antimicrobial peptide defences.

## Background

Global populations of frogs are declining and some species are facing extinction. One major cause is the fungal pathogen *Batrachochytrium dendrobatidis*, which causes chytridiomycosis [1–4]. The relationship between global climate change and increased prevalence of this disease is not direct [5], as higher environmental temperatures reduce mortality after experimental infection [6] and infected frogs may seek warmer temperatures in nature to improve defenses [7], suggesting that other factors play a role in the emergence of this disease.

Antimicrobial peptides made in cutaneous glands are secreted to the skin surface (the usual location of *B. dendrobatidis* infection). Antimicrobial peptides from some species of frogs that are susceptible to chytridiomycosis are nonetheless effective in killing *B. dendrobatidis* fungi *in vitro* [8], suggesting that these frog species ought to be resistant to chytridiomycosis. However, it has been shown that corticosteroids suppress antimicrobial peptide expression in skin of *Rana esculenta* (now *Pelophylax esculentus*) and *Xenopus laevis* [9, 10], as occurs with tracheal antimicrobial peptide in cattle [11]. Therefore, we and others have hypothesized that corticosteroid-induced reductions in the synthesis of these antimicrobial peptides may predispose frogs to chytridiomycosis.

In support of this hypothesis, levels of antimicrobial peptides were low in infected compared to uninfected frogs in a single population [12]. Further, experimental reduction of skin peptides and skin commensals exacerbated the response to *B. dendrobatidis* challenge [13]. Finally, frogs in geographic areas with endemic *B. dendrobatidis* infection have been shown to experience seasonal mortalities [14], suggesting that environmental factors may influence host susceptibility.

The present study was intended to address the broad hypothesis that spread and increased prevalence of chytridiomycosis is a manifestation of climate change-related alteration in environmental temperatures, with stress-induced impairment of antimicrobial peptide expression predisposing to fungal infection. This hypothesis is consistent with the observed geographic spread of *B. dendrobatidis*, if it is assumed that increased susceptibility of otherwise-resistant frog species could promote spread of infection. The specific objective was to measure the effect of glucocorticoid treatment on gene expression of cutaneous antimicrobial peptides that are known to inhibit the in vitro growth of *B. dendrobatidis*, using a frog species considered susceptible to chytridiomycosis (*Lithobates* [*Rana*] *pipiens*) [15]. The antimicrobial peptides brevinin-1P and ranatuerin-2P were studied because they both had previously been sequenced in *L. pipiens* [16, 17], and, individually, members of both the brevinin-1P and ranatuerin-2P families of peptides are known to inhibit *B. dendrobatidis* growth *in vitro* [17, 18]. Corticosteroid treatment was associated with a significant increase in brevinin-1P gene expression, which was most notable at 24–40 days of corticosteroid administration. Ranatuerin-2P expression followed a similar but non-significant trend. Thus, this treatment protocol including corticosteroid administration and frequent norepinephrine-induced secretion increased AMP gene expression in the skin of *L. pipiens* under these experimental conditions.

## Results

Frogs were injected with the corticosteroid methylprednisolone or saline every 48 hours for 40 days. Frogs in the corticosteroid and control treatment groups were of similar body weight on day 0 (*P* = 0.74, Student’s *t*-test) and gained weight over the 40-day course of the experiment. Maximal increases in body weight, 11.8 ± 1.4 g for the steroid treatment group and 14.9 ± 0.9 for the control treatment group, were not different between groups (*P* = 0.10, Student’s *t*-test). Apart from one frog with otitis interna, no other gross or histologic lesions were found.

**Fig. 1:**
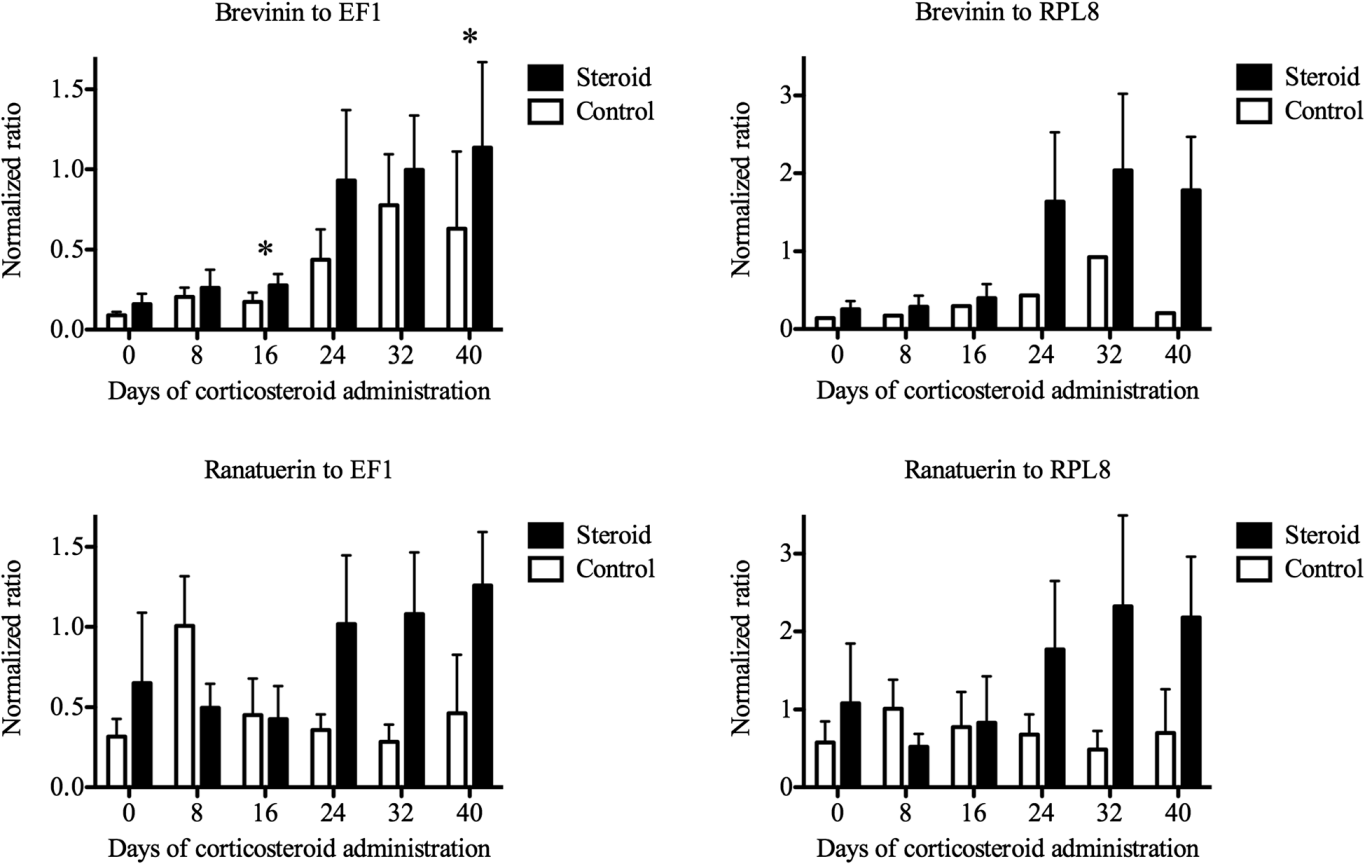
Brevinin-1P and ranatuerin-2P gene expression in norepinephrine-induced cutaneous secretions of frogs administered corticosteroid or saline. Comparison of steroid to control treatment groups, based on normalized ratios of the target gene (brevinin-1P or ranatuerin-2P) to the reference gene (EF1-α or RPL8) at the six sampling times (mean ± SEM, repeated measures ANOVA, *: significant at *P* < 0.05 between treatment groups)

**Fig. 2:**
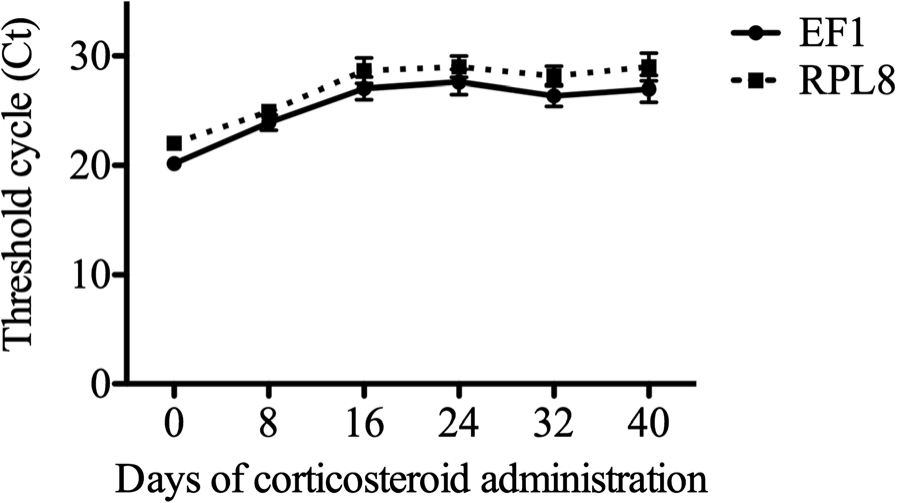
RT-qPCR threshold cycles of the two reference genes (EF1-α and RPL8) at the six sampling times during the experiment. EF1-α and RPL8 levels were significantly correlated (Pearson r = 0.965, *P* < 0.0001), and the levels changed significantly over time (mean ± SEM, repeated measures ANOVA)

Relative expression of two antimicrobial peptide genes was measured by RT-qPCR, in samples of norepinephrine-induced cutaneous secretions collected from corticosteroid- or sham-treated frogs, every 8 days for 40 days. Analysis of RT-qPCR data was based on the level of expression of each of the two target genes (the antimicrobial peptides brevinin-1P and ranatuerin-2P) relative to that of each of the two reference genes (RPL8, EF1-α) expressed as a ratio (Fig. 1). Over the six time points, the level of target gene expression appeared to increase within the steroid treatment group. For the brevinin-1P/EF1-α ratio, there was an overall significant (*P* < 0.027) increase in relative gene expression in the corticosteroid treatment group compared to the control treatment group (median [95 % confidence interval]: 0.08 [0.04, 0.14] and 0.28 [0.73, 0.11] respectively), with significant effect of treatment at 16 and 40 days. Although not statistically significant, an overall increase in gene expression in the corticosteroid treatment group was also found in the brevinin-1P/RPL8 ratio model (*P* =0.12), ranatuerin-2P/RPL8 ratio model (*P* =0.17), and ranatuerin-2P/EF1-α ratio model (*P* =0.11). RT-qPCR threshold cycles of the two reference genes were significantly correlated (Fig. 2) with no significant effect of corticosteroid treatment.

*Lithobates pipiens* expresses multiple closely related brevinin-1P and ranatuerin-2P genes [17] . To evaluate the specificity of the RT-qPCR assay, the sequences of the amplified products for ranatuerin-2P and brevinin-1P from 8 of the frogs were compared to sequences identified by mega-BLAST searches as well as for sequences identified in Genbank for ranatuerins/esculentins (*n* = 8) and brevinins (*n* = 26) from *L. pipiens*. The sequence of the ranatuerin RT-qPCR products matched only to ranatuerin-2P (AJ427747.1). The sequence of the brevinin RT-qPCR products contained variants at nucleotide position 38 (G/A) and 59 (C/T) relative to the reference sequence DQ276965. These variants matched to brevinin-1 Pa (DQ276965) and brevinin-1Pe (DQ276964) genes, as well as to the reported peptide sequence of brevinin-1Pc [17] but differed from the gene sequences of brevinin-1Pb, −1Pf, −1Pg, −1Pj, −1Pk or -1PLa. Thus, the RT-qPCR assays were specific for ranatuerin-2P, but amplified transcripts of more than one brevinin-1P gene.

## Discussion

This experiment investigated the effect of pharmacological concentrations of glucocorticoids on cutaneous gene expression of antimicrobial peptides that are known to inhibit the *in vitro* growth of *B. dendrobatidis*, in a frog species (*L. pipiens*) that is considered susceptible to this pathogen. We expected that exogenous glucocorticoid administration would inhibit the gene expression of these important peptides, supporting the hypothesis that glucocorticoid-induced suppression of this innate immune response may predispose to infection with *B. dendrobatidis*. Unexpectedly, the data showed the opposite effect, that corticosteroid administration was associated with increased antimicrobial peptide gene expression in the norepinephrine-induced cutaneous secretions of *L. pipiens*. Our protocol involved repeated maximal induction of peptide secretions [19] every eight days, and thus the population of cells producing peptides in the granular glands would have been rapidly producing mRNAs for defensive peptides to enable the frog to recover. Previous studies had suggested that pharmacological concentrations of corticosteroids would inhibit synthesis and renewal of defensive peptides [9, 10]. The present study suggests, instead, that corticosteroids may facilitate renewal of defensive peptides under these conditions.

*Lithobates pipiens* was studied because this species is known to produce two antimicrobial peptides that have anti-chytrid activity in vitro [17–19] and because it was reported to be susceptible to chytridiomycosis [15]. For example, this species suffered massive die-offs in the Colorado Rockies that were originally thought to be due to bacterial infection, but later analysis of museum specimens confirmed were due to chytrid fungal infection [18].

In this study, injection of norepinephrine resulted in visible accumulation of the holocrine secretions and permitted serial measurement of gene expression of the secreted antimicrobial peptides of interest. Amplification efficiencies for RT-qPCR were about 2.0, which is considered excellent. Threshold cycles for the reference genes—EF1-α and RPL8—were not affected by corticosteroid administration, and they were highly correlated with each other. Thus, this study establishes EF1-α and RPL8 as suitable reference genes for studies using RT-qPCR in this species.

Antimicrobial peptide gene expression was greater in norepinephrine-stimulated secretions from frogs that were administered corticosteroid, compared to frogs that were administered saline. This effect was statistically significant for the antimicrobial peptide brevinin-1P, using EF1-α as the reference gene. Although not statistically significant, a similar effect was seen for brevinin-1P using RPL8 as the reference gene, and for the antimicrobial peptide ranatuerin-2P relative to either of the reference genes.

Furthermore, this effect appeared to be time-dependent: the corticosteroid-treated frogs showed a trend of increasing levels of antimicrobial peptide gene expression over time, with significant effects at 16 and 40 days. The reason for this apparent time-dependence was not evident, and steroid administration was not associated with histologic changes in the glands. The timing of this effect was unexpected, because previously reported inhibitory effects of corticosteroids on antimicrobial peptide gene expression in cultured bovine tracheal epithelial cells were demonstrated at 40 h [11], and the inhibitory effects of corticosteroid on antimicrobial peptide levels in the skin of frogs were evident at 7 days of treatment in *L. esculenta* or 14 days in *X. laevis* [9, 10]. The reason for this delayed effect was not investigated, but we speculate that it may relate to an effect of corticosteroid on function of the granular glands that affected the level of antimicrobial peptide gene expression following depletion with norepinephrine.

Our findings contrast with those previously reported [9, 10], in which topical or systemic administration of glucocorticoids resulted in reduced gene expression of antimicrobial peptides in the skin of frogs. There are several methodological differences that may explain this discrepancy: the use of *L. pipiens* rather than *L. esculenta* or *Xenopus laevis*, the analysis of brevinin-1P and ranatuerin-2P rather than esculentin or total recoverable skin peptides as was shown for *X. laevis*, collection of secretions using norepinephrine rather than electrical stimulation, the use of saline-treated controls rather than untreated control frogs, and quantitation of gene expression using RT-qPCR rather than Northern blot. Other factors also may also have contributed to the different outcomes, including the nutritional status of the frogs, effects of sex hormones, the acclimatization period, the laboratory environment and housing, and the degree of handling stress. Nonetheless, the precise reasons for the discrepancy between the two studies were not determined.

Chronic exposure to corticosteroids is generally considered to be immunosuppressive, but shorter-term administration of corticosteroids augments some aspects of the innate immune response in mammals. For example, dexamethasone has several anti-inflammatory effects on neutrophils, but has been shown to increase neutrophil migration into the uterus [20] and lung [11]. Although psychological stress reduces cathelicidin and β-defensin expression in mice, it has been shown to increase expression of the antimicrobial neuropeptide catestatin [21] . Finally, glucocorticoids increase production of the surfactant proteins A and D by lung epithelial cells *in vitro*, and may increase the response to signalling through Toll-like receptors [22] . Thus, there is precedent for glucocorticoid-mediated enhancement of antimicrobial peptide defences. In support of these findings, a recent study found no evidence that corticosteroid administration exacerbated the response to experimental challenge with *Batrachochytrium dendrobatidis* [23] .

The findings of this *in vivo* study are subject to several limitations. First, it should be considered that handling stress or infections may have had an effect on antimicrobial peptide gene expression or modified the response to glucocorticoid administration. This possibility was mitigated by the long acclimatization period of 23 days, during which no overt evidence of infection was observed, and attention was paid to environmental enrichment conditions to minimize stress. Further, frogs were treated with an antibiotic prior to the study, and evidence of bacterial infection was not found at post-mortem examination. A second caveat is that the RT-qPCR method used in this study would not account for post-translational modifications that may affect secretion, function or half-life of the antimicrobial peptides.

## Conclusions

This study identified increased antimicrobial peptide gene expression in the skin of *L. pipiens* following treatment with the corticosteroid methylprednisolone. These findings suggest that corticosteroids do not impair and may even enhance cutaneous expression of antimicrobial peptides under the conditions of repeated peptide-depletion used in this study, and is inconsistent with the hypothesis that environmental stress predisposes frogs to chytridiomycosis by causing glucocorticoid-induced suppression of antimicrobial peptide defences.

## Methods

### Animals

Animal use was approved by the Animal Care Committee of the University of Guelph. Wild-caught northern leopard frogs (*L. pipiens*) were obtained from a commercial supplier. Thirteen frogs were separated arbitrarily into two groups (6 in the treatment group, 7 in the control group). The sex of the frogs was determined later at necropsy, with three males and three females in the treatment group, and two males and five females in the control group. Upon arrival, frogs were half-submerged in 100 mg/L oxytetracycline (Oxy-Vet 100LP, Vetoquinol, Quebec, Canada) for 1 h to prevent cutaneous bacterial infection.

The two groups of frogs were each held in plastic tanks of 1.1 m^2^ surface area, with terrestrial habitat made of potting soil, artificial plants and plastic structures for enrichment/hiding, and aquatic habitat consisting of 10 L of dechlorinated water 8 cm deep that was changed every three days. Frogs were free-fed live crickets, earthworms and mealworms in surplus every two days. Fluorescent lighting was on a 12:12 h abrupt-change photoperiod. Humidity was approximately 40 % and ambient temperature was 22-23 °C. The acclimatization period was 23 days.

### Study design

Frogs, identified by color pattern, were manually restrained using sterile nitrile gloves and each was weighed every 48 h. Starting on Day 0, an injection of either 0.075 mg/g body weight of the corticosteroid methylprednisolone (M0639, Sigma-Aldrich, Canada; treated group) or the same volume of 0.9 % saline solution (control group) was made into the dorsal lymph sac of the conscious frog every 48 h at the time of weighing. This dose and schedule were based on a prior study in *Lithobates esculenta* [9], as well as our unpublished observations.

Samples of norepinephrine-induced cutaneous secretions were used for sequential analysis of gene expression. Norepinephrine, an α-adrenergic agonist, stimulates discharge of the type I granular gland by a holocrine method of secretion with discharge of cellular mRNA [24, 25]. Skin gland secretions were sampled on Day 0 (after the initial steroid/saline injection), and then every 8 days until the end of the experiment at Day 40. Specifically, frogs were placed individually in sterile clear plastic bags and 40 nmol/g norepinephrine bitartrate (A0937-1G, Sigma-Aldrich, Canada) was injected into the dorsal lymph sac. Visible accumulation of secretions was noted within 3–5 min. After 10 min, the cutaneous secretions were collected into 300 μL of RNALater solution (Ambion, Austin, Texas), placed at 4 °C for 24–48 h, and then stored at −80 °C.

Frogs were euthanized using 100 mg/L benzocaine on Day 40 and a post-mortem examination was performed. Representative tissues were fixed in formalin, and hematoxylin- and eosin-stained histologic sections were routinely prepared and examined by a single blinded observer (LT).

### Measurement of antimicrobial peptide gene expression

Total RNA was isolated using an RNeasy Mini Kit (QIAGEN, Mississauga, Ontario) from cutaneous secretions collected and stored as described above. Samples were treated with DNase I Incubation Mix (QIAGEN, Mississauga, Ontario), and RNA yield and quality were analyzed by spectrophotometry at 260 and 280 nm (NanoDrop, Thermo Scientific, Wilmington, Delaware). The first-strand cDNA was synthesized using the Superscript III Reverse Transcriptase Kit (QIAGEN, Mississauga, Ontario). Samples were processed in batches of ten to fifteen. The cDNA synthesis was performed using a target of 100 ng total RNA, 1 μL Oligo (dT), 1 μL dNTP mix, and sterile distilled water to a total of 13 μL. Samples were incubated at 65 °C for 5 min. Then, 4 μL 5x first strand buffer, 1 μL 0.1 M DTT, 1 μL RNase OUT and 1 μL Superscript III RT were added, and samples were incubated at 50 °C for 1 h, followed by 70 °C for 15 min to inactivate the reverse transcriptase. Then, 1 μL RNase H was added to remove any residual RNA. Samples were incubated at 37 °C for 20 min, and stored at −20 °C.

RT-qPCR primers (Table 1) were based on available partial mRNA sequences for *L. pipiens* brevinin-1Pa (GenBank: DQ276965) and *L. pipiens* ranatuerin-2P (GenBank: AJ427747.1). EF1-α and RPL8 were used as reference genes, using previously published primers (for *Xenopus laevis*) for amplification of the coding regions (Table 1) [26, 27]. The RT-qPCR procedure was optimized using samples of skin from a healthy *L. pipiens* frog, initially using PCR, gel electrophoresis and sequencing of the amplified product. The amplified product was used to make the qPCR standard curves, using 12 serial dilutions from 10^−1^ to 10^−12^. A standard curve was generated for each primer set, performed in triplicate.

**Table 1:** Primers for RT-qPCR measurement of antimicrobial peptide gene expression

Gene	Forward Primer (5´-3´)	Reverse Primer (5´-3´)	Amplicon size (bp)	Efficiency
EF1-α (Reference gene)	CACACTGCTCACATTGCTTGC	ACAATGGCAGCATCTCCAGAC	209	2.07
RPL8 (Reference gene)	CACAGAAAGGGGCTGCTAAG	CAGGATGGGTTTGTCAATACG	254	2.09
Brevinin-1P (Target gene)	TGAAACGGATGTTGAAGTGG	GTGATTGCCATCTGGTGTGC	178	2.09
Ranatuerin-2P (Target gene)	CCAAAGATGTTCACCATGA	CATATGTCCGGCCAAATTCT	187	1.99

RT-qPCR analysis of the test samples was performed using 1 μL of cDNA (or PCR-grade water, for the negative control wells) added to a premix consisting of 5 μL SYBR Green 1 (Roche Applied Science, Eugene, Oregon), 0.5 μL of 20 μM of each forward and reverse primer, and 3 μL of PCR-grade water for a total volume of 10 μL. Real-time quantifications were performed using the Lightcycler 480 system (Roche Applied Science, Salt Lake City, Utah). Each assay was performed in duplicate with the following program parameters: pre-incubation at 95 °C for 5 min; amplification consisting of 45 cycles of denaturation at 95 °C for 20 s, annealing at 57 °C for 15 s, and elongation at 72 °C for 20 s; a melting curve from 45 to 95 °C; and cooling at 40 °C for 10 s. The fluorescence threshold value was calculated using Lightcycler 480 system software. The cDNA from one sample was used as a calibrator for each 96-well plate. Analysis of melting curves confirmed the consistency of the amplified product.

### Analysis of data

For analysis of RT-qPCR data, each of the two target genes (brevinin-1P, ranatuerin-2P) was compared individually to each of the two reference genes (EF1-α, RPL8) using the normalized values. The normalized values were defined as the ratio of the threshold cycle of the target gene to the reference gene, in relation to the calibrator value. Within each paired comparison, the means of the normalized values for the treatment group were compared to the means of the normalized values for the control group at individual time points.

A generalized linear mixed model was used to fit the data using Proc MIXED (SAS 9.2) with fixed effects of time, treatment and the covariates EF1-α and RPL8; plate was considered a random effect. Outcome variables included brevinin-1P and ranatuerin-2P gene expression levels in relation to each of the reference genes EF1-α and RPL8. There were repeated measures over time on individual frogs, so to handle this, various error structures were entertained and the error structures with the smallest Akaike information criterion were chosen (among those that converged, as offered by SAS: variance component, ar(1), arh(1), toeplitz, banded toeplitz, unstructured and banded unstructured error structures). All two-way interactions among time, treatment and the covariates were entertained, as were the three-way interactions between treatment, time and the covariates; in addition, the quadratics of EF1-α and RPL8 were also included in the model. Effects that were not significant with *p* > 0.05 were removed from the model except the main-effects primary factors of interest. Residual analyses were performed to examine the ANOVA assumptions. This included formally testing the residuals for normality using the four tests offered by SAS. In addition, the residuals were plotted against the predicted values and the explanatory variables used in the model. Such analyses may reveal outliers, unequal variances or the need for data transformation. ANOVA assumptions were adequately met.

## Abbreviations

EF1-α: Elongation factor-1α
RPL8: 60S ribosomal protein L8
